# Surface Chemical and Morphological Analysis of Chitosan/1,3-β-d-Glucan Polysaccharide Films Cross-Linked at 90 °C

**DOI:** 10.3390/ijms23115953

**Published:** 2022-05-25

**Authors:** Barbara Gieroba, Anna Sroka-Bartnicka, Paulina Kazimierczak, Grzegorz Kalisz, Agnieszka Lewalska-Graczyk, Vladyslav Vivcharenko, Robert Nowakowski, Izabela S. Pieta, Agata Przekora

**Affiliations:** 1 Independent Unit of Spectroscopy and Chemical Imaging, Chair of Biomedical Sciences, Medical University of Lublin, Chodzki 4a, 20-093 Lublin, Poland; barbaragieroba@umlub.pl (B.G.); grzegorzkalisz@umlub.pl (G.K.); 2 Independent Unit of Tissue Engineering and Regenerative Medicine, Medical University of Lublin, Chodzki 1, 20-093 Lublin, Poland; paulina.kazimierczak@umlub.pl (P.K.); vladyslav.vivcharenko@umlub.pl (V.V.); 3 Institute of Physical Chemistry Polish Academy of Sciences, Kasprzaka 44/52, 01-224 Warsaw, Poland; alewalska-graczyk@ichf.edu.pl (A.L.-G.); rnowakowski@ichf.edu.pl (R.N.); ipieta@ichf.edu.pl (I.S.P.)

**Keywords:** chitosan, 1,3-β-d-glucan, polysaccharide biomaterials, cross-linked polymers, structural analysis, surface properties

## Abstract

The cross-linking temperature of polymers may affect the surface characteristics and molecular arrangement, which are responsible for their mechanical and physico-chemical properties. The aim of this research was to determine and explain in detail the mechanism of unit interlinkage of two-component chitosan/1,3-β-d-glucan matrices gelled at 90 °C. This required identifying functional groups interacting with each other and assessing surface topography providing material chemical composition. For this purpose, various spectroscopic and microscopic approaches, such as attenuated total reflection Fourier transform infrared spectroscopy (ATR FT-IR), Raman spectroscopy, X-ray photoelectron spectroscopy (XPS) and atomic force microscopy (AFM), were applied. The results indicate the involvement mainly of the C-C and C-H groups and C=O⋯HN moieties in the process of biomaterial polymerization. Strong chemical interactions and ionocovalent bonds between the N-glucosamine moieties of chitosan and 1,3-β-d-glucan units were demonstrated, which was also reflected in the uniform surface of the sample without segregation. These unique properties, hybrid character and proper cell response may imply the potential application of studied biomaterial as biocompatible scaffolds used in regenerative medicine, especially in bone restoration and/or wound healing.

## 1. Introduction

Recently, there has been a growing interest in chitosan and glucan polymers because of their multiple applications, including tissue engineering, wound healing, medicine, immunology, and other branches of industry (cosmetics, textiles, food, agriculture) [1]. This popularity is due to their attractive and unique properties, including their biocompatibility, biodegradability, non-toxicity, and environmental friendliness. Moreover, they exhibit many favorable biological characteristics, such as antibacterial, antiviral, antifungal, antiparasitic, antioxidant, anticancer, cardiovascular, anti-inflammatory, and hepatoprotective activities [2]. In connection with desired biomedical features, scientific studies are currently focusing on creating biomaterials with the most beneficial parameters that will stimulate cells to grow and differentiate to form fully-developed and completely functional tissue [3]. Due to the fact that the outermost interface of the biomaterial directly interacts with the biological environment, the most important in this regard seems to be the surface properties that determine the initial adhesion of cells and then the proper cellular response. These features include surface hydrophilicity and charge resulting from chargeable functional groups (-OH, -CH_3_, -NH_2_, -COOH, -CH_2_OH, -CONH_2_ and -CH_2_NH_2_ groups), roughness (topography and pore size), softness and stiffness [4]. Surface analysis can significantly contribute to advancements in the optimization, manufacturing, and quality control of biomaterial production. Currently, predicting biological response on the basis of examined surface structure is a pivotal and dynamically developing field in surface research. The ATR mode of FT-IR sampling has been applied frequently in biomaterials studies [5]. The penetration depth of the infrared beam into the sample is commonly between 0.5 and 2 μm. Therefore, ATR is not only a highly surface-sensitive technique but also infiltrates a considerable region nearby the surface, thus giving information on a large sample area [6]. Raman and FT-IR spectroscopies are complementary techniques that are based on the measurement of vibrational and rotational energy changes. Raman spectroscopy constitutes a very useful tool in biomedical surface studies on account of its transparency to water. The characterization of water-containing biomaterials such as hydrogels is not an impediment in Raman spectroscopy, which is in contrast to FT-IR absorbing H_2_O radiation very strongly in the infrared range. However, low signal sensitivity, sample fluorescence, and thermal damage may pose a problem in Raman spectroscopy. However, these disadvantages are outweighed by the better detection of amino acid residues, S–S disulfide bridges, aromatic bonds, C–S, and C–C linkages in comparison with FT-IR spectroscopy, where additional difficulties arise from the overlapping bands [7]. Another spectroscopic technique, X-ray photoelectron spectroscopy (XPS), is particularly helpful in characterizing the elemental structures and surface chemistry (chemical functional groups) of biomaterials and is therefore called a surface-sensitive technique. X-rays are able to penetrate the sample surface up to many micrometers, but only a small part of the photoelectrons generated comparatively near the outside (~10 nm) have sufficient energy to escape into the vacuum system in a photoionization process. The photoelectric emission is the energy resolved to create a signature spectrum of electron intensity depending on the energy [8]. In turn, atomic force microscopy (AFM) enables the imaging of surfaces at very high (sub-nanometer) resolutions, and provides additional details concerning superficial mechanics and molecular interactions. It can be used to visualize single molecules, examine surface topography, and determine the forces that hold biological structures together. It is quite often utilized in biomaterial sciences in view of the comparatively slight interactions between the tip and visualized sample, mainly in the lateral direction of the surface [9].

In the present work, considering the above-mentioned analytical approaches and key physico-chemical features of biomaterials used in regenerative medicine, we have investigated the surface of chitosan/1,3-β-d-glucan matrices gelled at 90 °C by means of attenuated total reflection Fourier transform infrared (ATR FT-IR), Raman, and X-ray photoelectron spectroscopy (XPS) and atomic force microscopy (AFM). Our purpose was to check how these two polysaccharide components combine with each other at this gelation temperature. Although the use of vibrational spectroscopy to characterize chitosan [10] and 1,3-β-d-glucan-based materials [11] is not a new approach, the novelty of this examination is the intensive analysis of the spectra obtained in terms of attempting to quantify the data to provide the materials’ surface chemical composition depending on the gelation temperature of hybrid chitosan/1,3-β-d-glucan films. These studies are also an extension of our previous research on chitosan/1,3-β-d-glucan films cross-linked at lower, intermediate temperatures (70 °C and 80 °C), where we found a hybrid nature of polymeric matrices and stronger interactions at 80 °C [12,13]. In higher temperatures (up to 100 °C), curdlan forms a resilient gel; therefore, we predicted exceptional mechanical and chemical properties of the films subjected to 90 °C, such as greater strength resulting from strong ionocovalent bonds.

## 2. Results

### 2.1. Vibrational Spectroscopy

**Figure 1:**
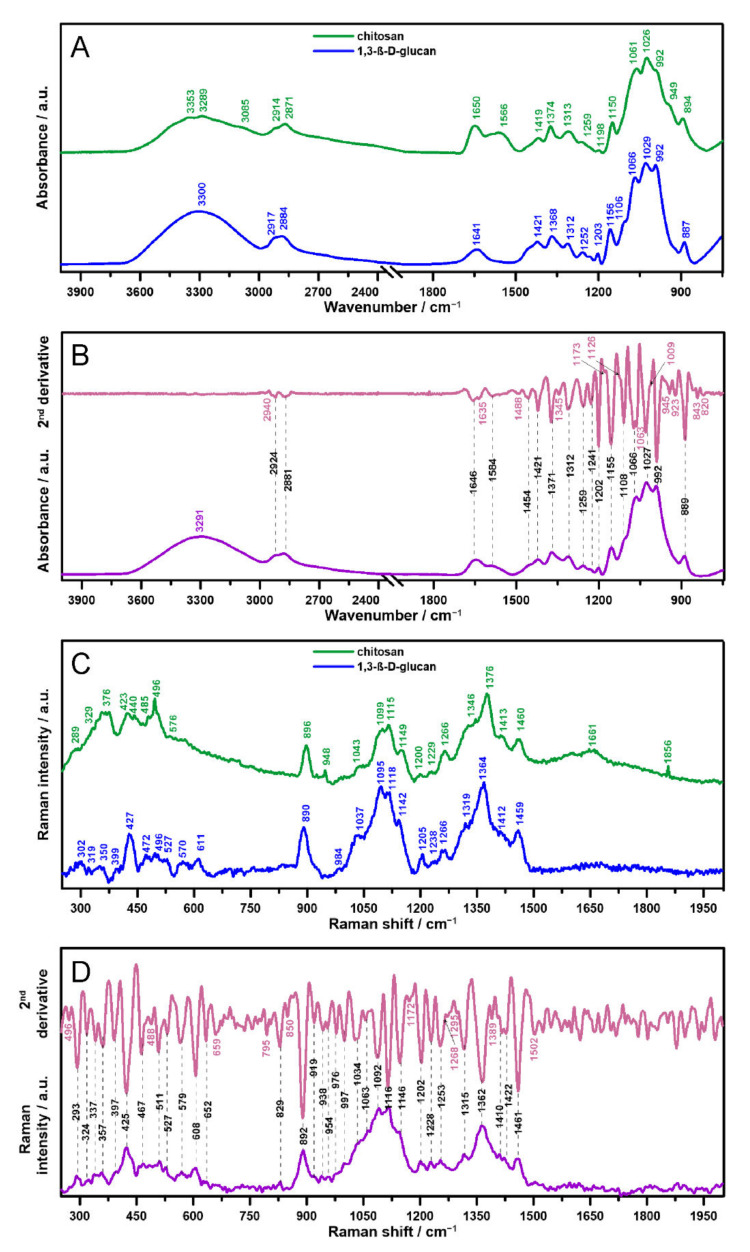
ATR FT-IR (**A**,**B**) and Raman (**C**,**D**) spectra of separate components of the polymeric matrix, gelled films of chitosan and 1,3-β-d-glucan (**A**,**C**); the relative intensity of hybrid chitosan/1,3-β-d-glucan spectra along with the second derivatives of the spectra in the entire measured range (**B**,**D**).

ATR FT-IR and Raman spectra of pure components (gelled films consisting of individual polymers of chitosan and 1,3-β-d-glucan individually) are presented in Figure 1A,C, respectively. Subsequently, spectra for chitosan/1,3-β-d-glucan films cross-linked at 90 °C were measured and shown in Figure 1B (ATR FT-IR spectroscopy) and 1D (Raman spectroscopy), along with their second derivatives. The most important bands in the vibrational spectra and the minima in the course of the second derivatives are assigned wavenumbers.

Table 1 and Table 2 contain a list of the most important bands in vibrational spectra, with corresponding assignments presented in Figure 1A,B (ATR FT-IR spectra, Table 1) and Figure 1C,D (Raman spectra, Table 2) [12,14,15,16,17]. Both ATR FT-IR and Raman spectra of the individual components, chitosan and 1,3-β-d-glucan (Figure 1A,C, respectively), allow for the unambiguous identification of these compounds and are consistent with the literature data [11,18]. Spectroscopic investigation of multicomponent materials, where many covalent and ionic bonds are formed between and within the individual components, is a little more complex and often not obvious. Moreover, analysis of ATR FT-IR and Raman spectroscopic data becomes intricate since the bands often overlap, and, thus, second derivative determination enhances their separation [19]. Therefore, additional bands in chitosan/1,3-β-d-glucan spectra highlighted in pink were obtained on the basis of the second derivatives. In the ATR FT-IR spectrum (Figure 1B), a second derivative minimum at 2940 cm^−1^ is ascribed to C–H stretching [20]; 1635 cm^−1^ is ascribed to a β-sheet secondary structure in the amide I range [21], 1488 cm^−1^ is ascribed to aromatic C=C stretching, 1345 cm^−1^ is ascribed to OH deformation, 1173 cm^−1^ is ascribed to C–C ring and C–N stretching and OH and CH deformation, and 1126 cm^−1^ is ascribed to C–N ring stretching and CH deformation. In turn, 1063 cm^−1^ is assigned to CH_2_ wagging from CH_3_ groups, 1009 cm^−1^ is assigned to C–C ring, C–N ring and N–O stretching, 945 cm^−1^ is assigned to CH and OH deformation, 923 cm^−1^ is ascribed to CH_2_ rocking, 843 cm^−1^ is assigned to C–N stretching and CH_2_ rocking, and 820 cm^−1^ is ascribed to C–H, OH, and C=O deformation [20]. In the Raman spectrum (Figure 1D), 496 488, and 659 cm^−1^ s derivative minima are attributed to C–C bending, 795 cm^−1^ is ascribed to CH_2_ rocking, 850 cm^−1^ is assigned to =C–H wagging, 1172 cm^−1^ is assigned to CH_3_ wagging, 1268 cm^−1^ is ascribed to CH_2_ twisting, 1295 cm^−1^ is ascribed to =C–H bending, 1389 cm^−1^ is attributed to CH_3_ asymmetrical deformation, and 1502 cm^−1^ is attributed to CH_2_ deformation [22]. The Raman results indicate the formation of links between the polymer chains, which probably form a tightly packed and rigid structure.

The ATR FT-IR and Raman spectra allow the differences in the molecular structure of the studied compounds to be demonstrated. Based on the measured spectra and assigned appropriate functional groups, it is possible to detect chemical interactions between chitosan and 1,3-β-d-glucan in the process of creating polymer matrices at a temperature of 90 °C. In the vibrational spectra of chitosan, OH groups of glucopyranose units, NH and NH2 groups were detected, which were not recorded in the 1,3-β-d-glucan and chitosan/1,3-β-d-glucan spectra. Moreover, fewer bands arising from C–C and more from C=C bonds were observed; a different pattern of C–C–C, C–C–N vibrational modes, and a lack of C–C(=O)C groups were found. In turn, in the 1,3-β-d-glucan spectra, a different mode of C–H vibrations and lack of an amide II band were the most pronounced findings. In the chitosan/1,3-β-d-glucan sample, additional bands ascribed to C–H stretching in rings and CH_3_ and CH_2_ symmetrical bending vibrations emerged. The CH_3_ functional groups were presumably related to the inherence of N-acetyl-glucosamine units. Additionally, bands attributed to O–C–C, C–O–C, C–C=O, and C–N=C appeared especially visible in the Raman spectra. These observations indicate the presence of a chemical interaction between interactions between the rings of both components: chitosan and 1,3-β-d-glucan polymers, and interconnections, especially between the –NH group of N-acetylglucosamine and/or glucosamine units of chitosan and probably the –OH group of the glucan units in 1,3-β-d-glucan [23]. Based on our previous research [12], it can be assumed that this interlinkage is initiated and intensified by a high temperature of gelation (90 °C) and testifies to the hybrid nature of chitosan/1,3-β-d-glucan matrices.

More precisely, comparing the results of vibrational spectroscopy studies regarding the cross-linking pattern of the samples gelled at 90 °C and at intermediate temperatures of 70–80 °C, significant differences can be noticed [12]. Direct comparison is available in Table S1 in Supplementary Materials. At higher temperatures in ATR FT-IR spectra, there was no band at 2924 cm^−1^ ascribed to CH_2_ stretching vibrations, affecting more chitosan moieties to create C-H vibrations in rings. Moreover, the bands at 1241 cm^−1^ related to the C–H stretching vibrations in the rings (also not observed in single components spectra) and at 1108 cm^−1^ assigned to C–C and C–O stretching modes appeared. The shift of the amide II band towards higher wavenumbers (1566 cm^−1^ in samples gelled at 70 °C and 80 °C, and 1584 cm^−1^ in samples gelled at 90 °C) is also clearly visible. A noticeable blue-shift in the amide II of intermediate temperature gels indicates a lower ratio of intramolecular hydrogen bonds to stronger intersheet hydrogen bonds of the resulting polymer film in the 90 °C sample [24]. In turn, in the Raman spectra at 90 °C, there were bands at 467, 562, 919, 976 cm^−1^ attributed to N–C=O and C–O–C, C–C=O bending, C–O–C and ring skeleton stretching vibrations, respectively. Additionally, the bands at 1410 and 1422 cm^−1^ arising from CH_3_ deformations and C–C stretching emerged. The disappearance of bands at 1502 and 1620 cm^−1^ connected with C–C and C=O stretching modes is also noteworthy. Furthermore, slight shifts of some bands were recorded. All these observed changes suggest the formation of additional linkages between the chitosan and 1,3-β-d-glucan subunits at higher studied temperatures and testify to the stronger interactions and tighter packing of these matrices [12].

**Figure 2:**
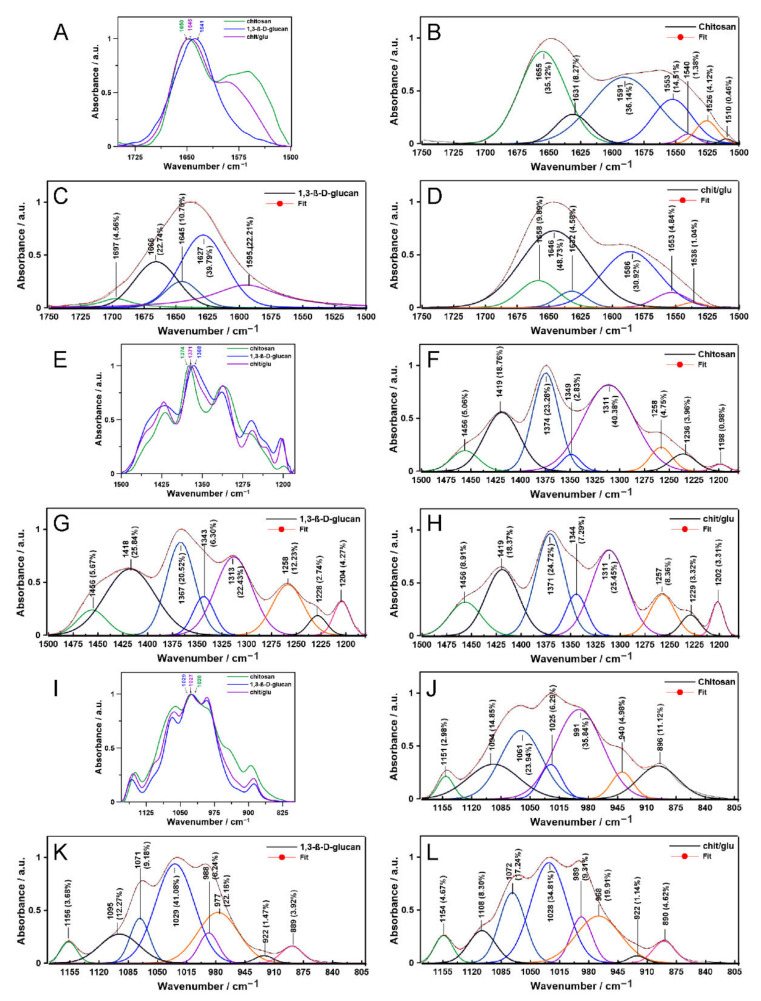
The normalized ATR FT-IR absorbance (**A**,**E**,**I**) spectra in the selected ranges and band deconvolution with the application of mixed Lorentzian/Gaussian curve fitting (**B**–**D**): 1750–1500 cm^−1^, (**E**–**H**): 1500–1180 cm^−1^: (**J**–**L**): 1180–800 cm^−1^. Each subband has marked a maximum value and a percentage share of the whole band surface area.

**Figure 3:**
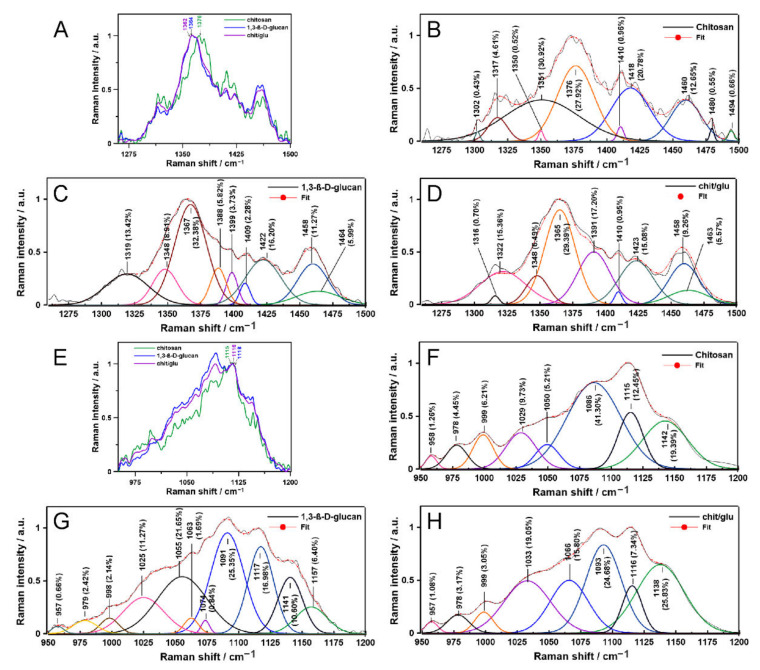
The normalized Raman intensity (**A**,**E**) spectra in the selected ranges and band deconvolution with the application of mixed Lorentzian/Gaussian curve fitting ((**B**–**D**): 1260–1500 cm^−1^, and (**F**–**H**): 950–1200 cm^−1^). Each subband has marked a maximum value and a percentage share of the whole band surface area.

The spectra enable the qualitative detection of chemical groups together with vibrational modes in the samples, while the semi-quantitative analysis requires their normalization and deconvolution, as shown in Figure 2 and Figure 3. The normalized ATR FT-IR spectra in the amide I and II region (1750–1500 cm^−1^), ranges characteristic for carbonyl, the CH, CH_2_, and CH_3_ groups (1500–1180 cm^−1^), and a fingerprint region including a specific marker for carbohydrates (1180–800 cm^−1^) [25] were shown in Figure 2A,E,I, respectively. In turn, the normalized Raman spectra in the range including the CH, CH_2_, CH_3_ modes and NH in-plane vibrations (1260–1500 cm^−1^) [26] and 950–1200 cm^−1^ region representative for C–C, C–O–C and C–N stretching and skeletal vibrations [27] were presented in Figure 3A,E, respectively.

In the 1750–1500 cm^−1^ ATR FT-IR spectral range (Figure 2A), most evident is the absence of an amide II band for the 1,3-β-d-glucan. Instead, a small percentage band occurs at 1697 cm^−1^, which can be assigned to C=O stretching coupled to the NH_2_ scissoring and N–H in-plane bending vibrations (Figure 2C) [28]. Compared to the 1,3-β-d-glucan, in the spectrum of chitosan/1,3-β-d-glucan matrices, a band at 1645 cm^−1^ related to C=O carboxylic groups and C=C stretching vibration of rings [29] shifts to 1646 cm^−1^ and a band at 1627 cm^−1^ derived from stretching of the C=O group [29] shifts to 1632 cm^−1^. In turn, compared to chitosan, in the spectrum of chitosan/1,3-β-d-glucan polymers, the band at 1591 cm^−1^ ascribed to C–O vibrational bending [30] shifts to 1586 cm^−1^, and the band at 1553 cm^−1^, mainly related to in-plane amine N–H bending coupled to amine C–N stretching vibrations of amines [31], is at the same wavenumbers (Figure 2D). In chitosan (Figure 2B), a band with a minimal percentage share occurs at 1510 cm^−1^, corresponding to the stretching asymmetric vibrations of the C–N group [32]. In the 1500–1180 cm^−1^ infrared range (Figure 2E), chitosan/1,3-β-d-glucan (Figure 2H) and 1,3-β-d-glucan (Figure 2G) show a similar course of deconvolution, with only slight shifts and a modest percentage of differences. The profile of deconvolution in the case of chitosan (Figure 3F) differs insignificantly; here, a larger share of the 1311 cm^−1^ band attributed to the stretching vibration of N–H, C–N and C–O for the amide III bond and a smaller share of the 1349 cm^−1^ band assigned to the CH_2_ wagging mode [33] is observed. In the 1200–800 cm^−1^ FTIR range (Figure 2I), the situation is similar; spectral deconvolution for the 1,3-β-d-glucan (Figure 2K) and the chitosan/1,3-β-d-glucan (Figure 2L) has a comparable course and is characterized by the different pattern for the chitosan sample (Figure 2J). There is a smaller share and a shift towards the lower wavenumber of the band at 1025 cm^−1^ and a much larger share of the band at 991 cm^−1^; these two regions are associated with the C–O stretching mode [34]. There is also no 922 cm^−1^ band here connected with the C–H deformation of β-glycosidic linkages, but there is an additional 940 cm^−1^ band assigned to the O–H out-of-plane deformation [34].

In the 1300–1500 cm^−1^ Raman range (Figure 3A), chitosan/1,3-β-d-glucan deconvolution is the combination of both chitosan (Figure 3B) and 1,3-β-d-glucan (Figure 3C) profiles. Noteworthy is the broadened band at 1351 cm^−1^ in chitosan, accounting for almost 31% of the area of all bands, and an additional small band at 1302 cm^−1^ attributed to CH_2_ wagging and the CH deformational [35], respectively (Figure 3B). In the 1000–1200 cm^−1^ Raman region (Figure 3D), the deconvolution pattern shows a characteristic broadening at 1086 cm^−1^ (chitosan, Figure 3F), 1055 cm^−1^ (1,3-β-d-glucan, Figure 3G) and 1138 cm^−1^ (chitosan/1,3-β-d-glucan, Figure 3H) associated with C–N stretching, C–O stretching and C–H deformational vibrations, respectively [36]. In the 1,3-β-d-glucan profile, there are two bands at 1063 and 1074 cm^−1^ (Figure 3G), which disappear in chitosan/1,3-β-d-glucan (Figure 3H), forming one peak at 1066 cm^−1^ connected with C–C stretching and C–H bending [36]. The ATR FT-IR and Raman results show that C=O⋯HN moieties in the N-acetylglucosamine groups of chitosan are particularly involved in the polymerization process with 1,3-β-d-glucan units.

Based on the data obtained from the ATR FT-IR and Raman spectra, the molecular structure of polymeric matrices can also be evaluated semi-quantitatively by calculations of the FT-IR absorbance and Raman intensity coefficients for the most significant chemical groups involved in the cross-linking process. Table 3 presents the compilation of the acquired ratios. Before the analysis, the spectra were normalized to increase the analysis’s accuracy.

The semi-quantitative differences between the components of the individual samples can be traced more closely using both the ATR FT-IR absorbance and Raman intensity ratios (A_amide I_/A_amide II__,_ A_CH_2__/A_CH_3__, A_C=O_/A_C–O_, I_CH and/or C–OH_/I_CH_3___,_ and I_C–O–C_/I_C–C_). The ATR FT-IR absorbance ratio between amide I and amide II derived from C=O and N–H stretching vibrations of amide groups can be applied to assess the changes in the secondary structure of the chains [37] of studied polysaccharide polymers. Due to the lack of an amide II band in the 1,3-β-d-glucan, this ratio has not been calculated for this sample. The amide I/amide II coefficient is higher in the chitosan/1,3-β-d-glucan matrices than in chitosan, which may indicate a different molecular arrangement of the chains resulting from the occurrence of different interactions in the mixed sample. Considering the methyl/methylene groups ratio (A_CH_2__/A_CH_3__), it can be concluded that it is the lowest in 1,3-β-d-glucan and comparable in chitosan and chitosan/1,3-β-d-glucan samples. The CH_2_/CH_3_ absorbance ratio is a suitable indicator of aliphatic chain length and branching. Even though the CH_2_/CH_3_ coefficient does not correspond to the molar abundance ratio of CH_2_/CH_3_ caused by the variation of molar absorption coefficients between CH_2_ and CH_3_, the CH_2_/CH_3_ infrared absorbance ratio demonstrates a linear correlation with the factual number of CH_2_/CH_3_ in polymers [38]. The analysis of the A_C=O_/A_C–O_ ratio shows that it is the highest in 1,3-β-d-glucan, the lowest in chitosan and intermediate in the chitosan/1,3-β-d-glucan specimen. This allows the evaluation of the number of carbonyl groups with respect to the C–O groups, which indicates how interchain bonds are formed [39,40]. I_CH and/or C–OH_/I_CH__3_ obtained from the Raman spectra demonstrate that this ratio is the highest in 1,3-β-d-glucan, which may indicate that the connections between the polymer units are more similar in chitosan and chitosan/1,3-β-d-glucan while differing in 1,3-β-d-glucan. The CH stretching vibration corresponds to the pyranoid ring, and this intensity ratio relates to the changes in the degree of N-acetylation [41]. The I_C–O–C_/I_C–C_ ratio is the lowest in chitosan and the highest in chitosan/1,3-β-d-glucan. The C–O–C vibrational modes may be present within rings at the chain linkages and the branching points [42]. The C–O and C–C stretching modes are characteristic of the glycosidic linkage attached to the glucosyl unit. It originates from the vibrations of the C–O–C moiety [43]. Differences in this coefficient may suggest another type of unit inter-connection in the chains.

### 2.2. XPS Spectroscopy

**Figure 4:**
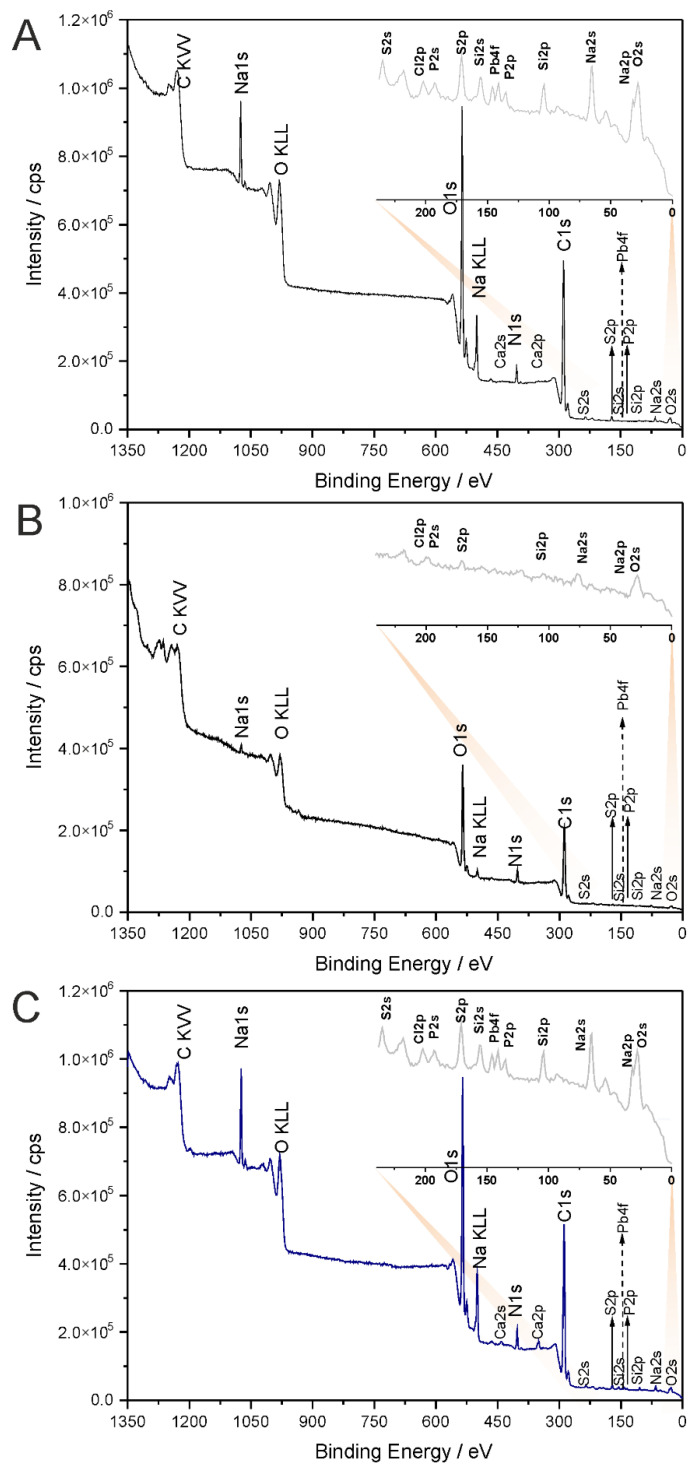
XPS wide scan spectra for (**A**) glucan, (**B**) chitosan and (**C**) hybrid chitosan/1,3-β-d-glucan samples gelled at 90 °C.

**Figure 5:**
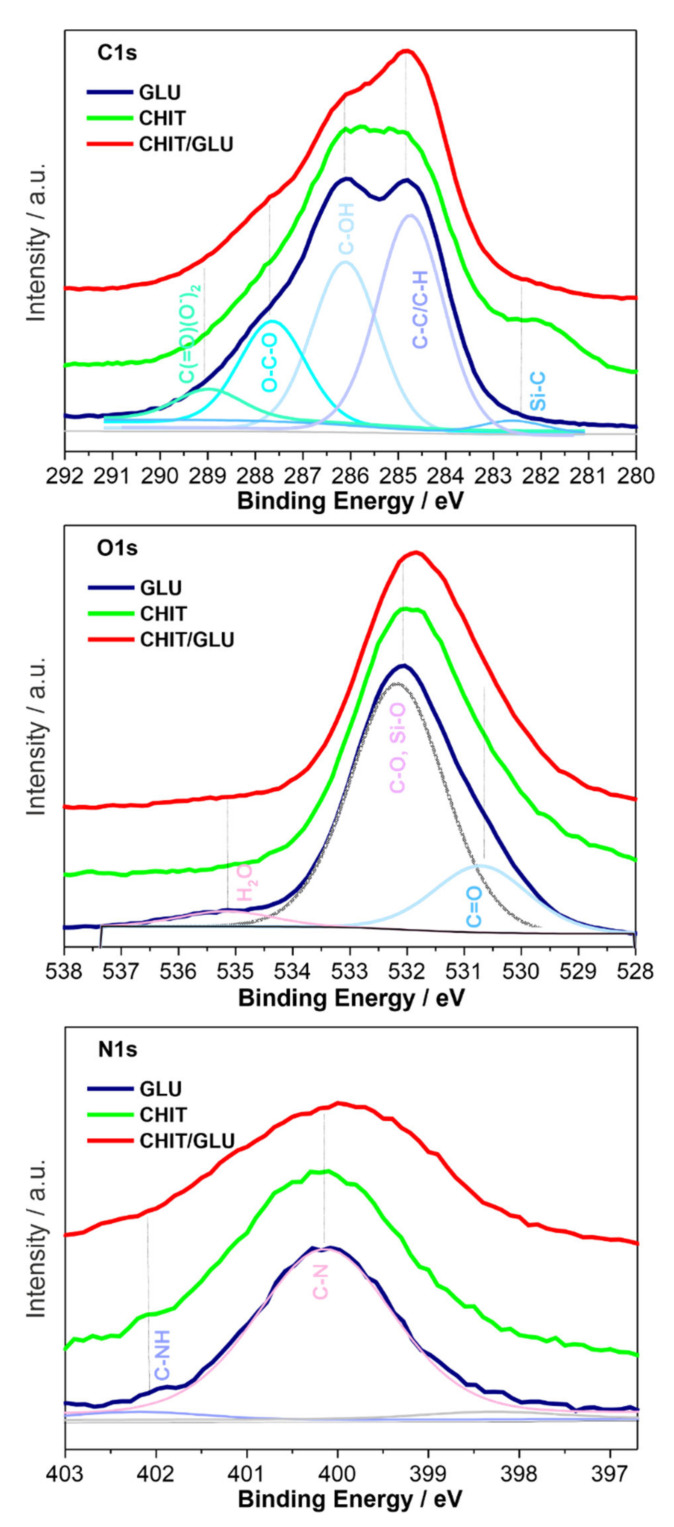
XPS C1s, O1s and N1s core-level spectra for glucan (GLU), chitosan (CHIT) and hybrid chitosan/1,3-β-d-glucan (CHIT/GLU) samples, with peak decomposition and component position and assignment.

The molecular structure of pure chitosan and 1,3-β-d-glucan and hybrid chitosan/1,3-β-d-glucan matrices were elucidated by means of vibrational spectroscopy. X-ray photoelectron spectroscopy was used to determine the chemical state of the surface components in the hybrid specimen. Figure 4 presents the full survey spectra of glucan, chitosan and hybrid chitosan/β-1,3-glucan samples gelled at 90 °C, while corresponding core-level spectra for C1s, N1s, and O1s regions are shown in Figure 5. Table 4 presents the calculated chemical composition of polymeric samples.

The investigated glucan, chitosan and hybrid chitosan/1,3-β-d-glucan samples predominantly contained C, N and O with minor contributions of S, Na, Cl, Ca, P and Si. Table 4 shows the chemical contents calculated for the polymer samples. In accordance with our previous study, the C:N (c.a. 13) and C:O (2.3) ratios indicated that the hybrid chitosan/1,3-β-d-glucan surface was enriched in C atoms [12]. Moreover, the XPS results show substantial changes in the molecular composition of gelled polymers. The C, N, and O atomic concentrations drop in the hybrid chitosan/1,3-β-d-glucan sample. At the same time, the S, P, Cl, and Ca surface concentrations are higher than those for corresponding monomeric samples, i.e., glucan and chitosan (Table 4).

In Figure 5, C1s, O1s, and N1s core level spectra are shown for all polymeric samples treated at 90 °C. For the glucan sample, the C1s signal was deconvoluted to five peaks with binding energies of 282.4, 284.6, 286.3, 287.8, and 289.1 eV, indicative of Si-C-O, hydrocarbon (C-H/C-C), alcohol/ether/amine (C-OX), acetal (O-C-O), and carbonyl-containing groups [44]. For the chitosan and the hybrid chitosan/1,3-β-d-glucan samples, all the signals observed in the glucan sample were also observed. The acetal (O-C-O)-related peak is observed in all samples, indicating that there is a small degree of change in the surface functionalization within the studied materials. However, compared to monomeric samples, the hybrid chitosan/1,3-β-d-glucan signal can be attributed to C-H/C-C and presents the highest intensity, confirming the strong envelopment of C-C and C-H bonds in the hybrid sample. According to our previous finding, the change in surface functionalization is more visible for the polymeric sample treated at different temperatures [12].

The profiles of the N1s and O1s peaks in the spectra of all investigated samples (Figure 5) are similar, indicating certain similarities within the type and the distributions of the most abundant surface species in these materials. As was suggested previously, this fact is most likely determined by the surface structure and topology, which may promote the formation of certain surface species/functions to balance the surface charge of the system [12]. This data agrees with the spectroscopic results, which indicate that the chitosan/1,3-β-d-glucan matrices caused modifications in the molecular arrangement and are consistent with spectroscopic observations showing that the hybrid chitosan/1,3-β-d-glucan samples reveal alterations in the molecular configuration.

### 2.3. AFM Imaging

**Figure 6:**
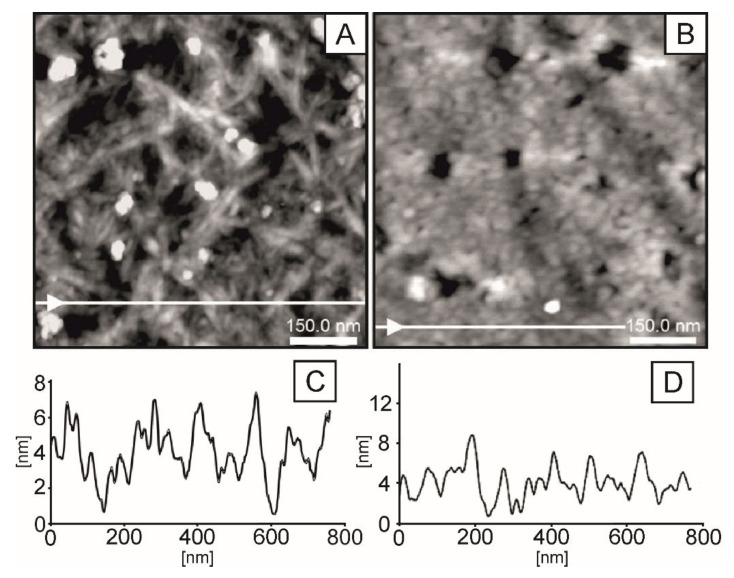
AFM images (**A**,**B**) and line profiles (**C**,**D**) of β-1,3-d-glucan (**A**,**C**) and chitosan (**B**,**D**) polymeric films. Scanning area: (**A**,**B**) 750 × 750 nm.

AFM images of investigated samples are depicted in Figure 6.

In the images presented in Figure 6, parts (A), (C) correspond to the glucan sample thermally gelled at 90 °C. Parts (B) and (D) correspond to the chitosan sample gelled in NaOH and subjected to high temperatures (90 °C). The production conditions were the same as for the chitosan/glucan sample. Both images show developed morphology. The glucan sample is characterized by a fibrous structure. In these cases, single fibres are easily distinguished on the AFM images as bright lines. The width of the observed longitudinal structures is in the range of 12–18 nm; however, this dimension can be overestimated by the shape of the AFM tip. The fibres are randomly distributed and mutually intertwined.

As a consequence, the surface of the glucan samples is relatively rough. They are additionally decorated by randomly distributed grains of the size between 50 and 70 nm and high 5–10 nm (visible in the images as bright spots). In contrast, the chitosan samples are characterized by different grain-like morphology. Image (B) and the corresponding line profile (D) indicate that in the majority, the size of grains is in the range of 30–40 nm (measured in horizontal dimension); however, larger grains of 110 nm in diameter are also locally observed. The grains in the film are densely packed, which leads to a lower surface roughness compared to the glucan layer (compare the lines of representative profiles (C) and (D)). In summary, the presented comparison clearly indicates a significantly different topology of glucan and chitosan samples. Therefore, an interesting question arises about the organization of hybrid samples consisting of both components.

**Figure 7:**
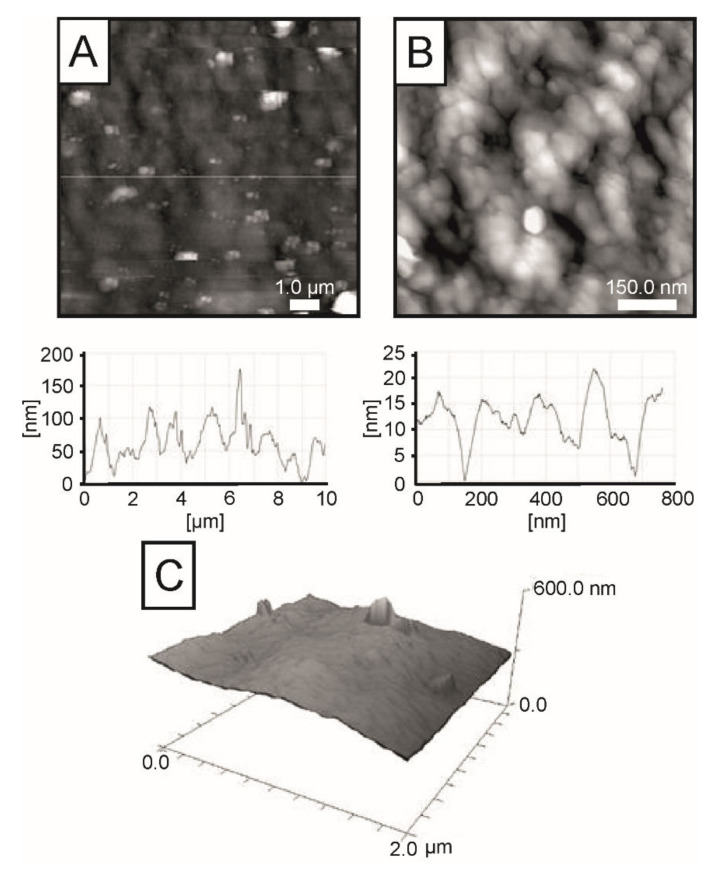
2D AFM images (**A**) 10 × 10 µm, and (**B**) 760 × 760 nm with corresponding line profiles and (**C**) 3D AFM image (2 × 2 µm) of chitosan/1,3-β-d-glucan sample gelled at 90 °C.

In Figure 7, the AFM images of the chitosan/1,3-β-d-glucan sample are presented (image size: (A) 10 × 10 µm and (B) 760 × 760 nm, (C) 2 × 2 µm). The characteristic features of this layer’s topography occur at two different scales. The main feature is the granular structure of the layer (Figure 7B), qualitatively similar to that observed for the chitosan layer. Despite the fact that single larger linear structures can be observed very locally on the surface, the film is dominated by randomly distributed and well-resolved grains. Important observational concerns are the grain size, whose average value is larger (approx. 110 nm) in comparison to grains of the chitosan film (see line profile in Figure 7B). As a consequence, the surface of the hybrid film is rougher, comparable to that observed for the glucan samples (compare line profiles presented in Figure 6C and Figure 7B). The second features are larger, randomly distributed protrusions additionally present on surfaces and are observed as bright spots on the larger-area images (Figure 7A). Their size (up to 175 nm) is noticeably larger than the average size of grains of the base surface. However, it is important to emphasize that the density of ad-species is very low for this temperature of the sample preparation. The 3D image additionally presented in Figure 7C confirm these observations. The presented AFM study indicates that the hybrid material is uniform and homogeneous, and no surface segregation occurs during film preparation.

### 2.4. Cytotoxicity Evaluation

**Figure 8:**
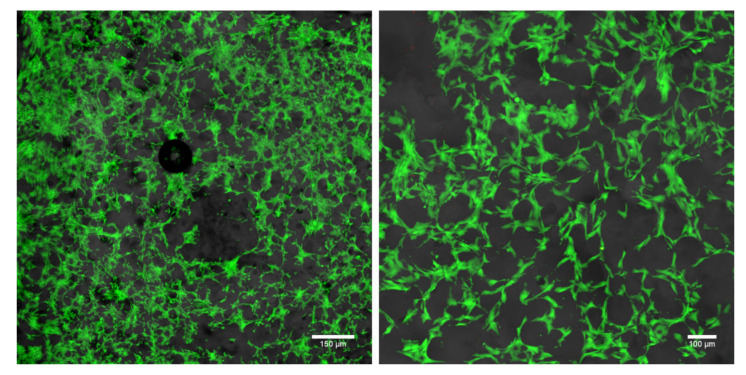
Confocal laser scanning microscope (CLSM) images presenting live/dead staining of BJ cells cultured on the chitosan/1,3-β-d-glucan film for 48 h (live cells—green fluorescence, dead cells—red fluorescence, Nomarski contrast was used to show the structure of films; magn. 40×, scale bar = 150 μm; magn. 100×, scale bar = 100 μm).

A cytotoxicity assessment of the produced chitosan/1,3-β-d-glucan film was performed by using live/dead staining of human skin fibroblasts (BJ cell line) cultured on the surface of the film for 48 h. Obtained confocal laser scanning microscope (CLSM) images showed a great number of viable cells (green fluorescence) (Figure 8). The cells were well flattened and attached to the surface of the produced film, confirming its non-toxicity.

## 3. Discussion

Generally, characterization is a substantial field in material science, allowing optimization of the material performance depending on its potential application. It concerns the determination of the physical and chemical properties of the material for a better understanding of its molecular structure. ATR FT-IR, Raman, X-ray photoelectron spectroscopies, and atomic force microscopy are recognized as potent analytical tools for investigating biological and chemical samples due to their label-free nature, little or no sample preparation requirements, along with the wealth of (bio)chemically specific information they provide [45]. Recently, spectroscopic and microscopic approaches have been used extensively for the analysis of biological and biochemical samples in vitro. However, there is a limitation to how much chemically specific information can be drawn out from a spectrum and microscopic image of a polymeric multicomponent sample consisting of multiple overlapping peaks from many compounds in any specific sample [46].

The fundamental difficulty encountered in analyzing such complex spectral data arises since the width of each component band is usually greater than the distance between the maximum adjacent bands; the individual elements cannot be separated and/or identified over a wide range of experimentally measured spectra. The extraction of the structural information encoded in these FT-IR and Raman bands requires extensive mathematical analysis of the experimental data [47].

In order to increase the separation of overlapping bands, the second derivative (SD) of the absorption spectrum is calculated, either in the frequency domain or by mathematical manipulation in the Fourier domain [48]. It should be noted that the second derivative of the spectrum does not maintain the relative intensities of the absorption bands. They depend on the width of each absorption in the original spectrum, so narrow bands will be amplified at the expense of wider bands [47,49].

The second derivative of the spectra is very sensitive to any interferences (such as instrumental noise, uncorrected water vapor absorption bands, etc.); therefore, its shape should be analyzed carefully. The minima of the second derivatives show the probable positions of the component bands that can be assigned to the individual structures. The advantage of the deconvolution method over the second derivative determination is that it introduces less distortion. In particular, it does not affect the integrated intensities (areas) of the individual elements of the bands [50]. Therefore, on the basis of the second derivative, the deconvolution procedure was carried out.

For deconvolution, it is important to define two parameters: full width at half-height (FWHH) and the resolution enhancement factor (peak sharpening). Their selection determines the number and maximum of the resulting sub-components. It is a critical operation that determines the quality of the results for a curve fitting procedure [51].

In an attempt to elucidate more precise data regarding molecular arrangement, we used ratiometric and deconvolutive points of view, for which it was also essential to determine the second-order derivative. These operations made it possible to accurately trace the molecular structure of the investigated polysaccharide polymers. In this study, we focused on the specific cross-linking conditions, particularly at the temperature, which was 90 °C, of 1,3-β-d-glucan, and the hybrid chitosan/1,3-β-d-glucan films. Chitosan films as control material for the hybrid matrices were gelled in NaOH and then subjected to 90 °C in order to create identical production conditions as mixed chitosan/1,3-β-d-glucan films.

Although the use of vibrational spectroscopy to characterize chitosan [10] and 1,3-β-d-glucan-based materials [11] is not a new approach, the novelty of this examination is the intensive analysis of the spectra obtained in terms of attempting to quantify the data to provide a material’s surface chemical composition depending on the gelation temperature of the hybrid chitosan/1,3-β-d-glucan films. Thus far, mixed chitosan/1,3-β-d-glucan was checked for antimicrobial properties [52], cell adhesion, growth and proliferation [53], wound healing in type 2 diabetic mice [54], and its impact on body weight and composition [55], but the molecular order, surface properties and functional groups involved in the formation of such matrices have not yet been studied in such depth. Studies concerning chitosan/1,3-β-d-glucan membranes for antibacterial applications conducted by Sun et al. showed that at 90 °C, a symmetrical blend membrane was formed; this was in contrast to 60 °C, where the phase separation was observed in SEM images. They also proved by means of FT-IR and differential scanning calorimetry (DSC) measurements that the interlinkages between the amino group of chitosan and the hydroxyl group of 1,3-β-d-glucan in polymeric matrices are easier to form when the cross-linking temperature is higher, and this may result in more intense intermolecular hydrophobic interactions. Under these conditions of preparations, it is most likely that an irreversible gel is created, determining its remarkable permeable and mechanical characteristics [52]. This is in line with our results because we also demonstrated a strong interplay between components at 90 °C and the formation of the homogenous and uniform surface of matrices. Formerly, we examined intermediate gelation temperatures (70 °C and 80 °C) and also proved more intense interactions at 80 °C [12]. It can therefore be concluded that the strength of the interaction increases with the temperature of cross-linking between the chitosan and 1,3-β-d-glucan units. We found that the chains in the chitosan/1,3-β-d-glucan film structure are held together by C=O⋯OH and C=O⋯HN bonds between the amide groups and by the -CH_2_OH side chains, which results in the creation of intersheet H-bonds to the carbonyl oxygens on the adjacent chains. This probably leads to a structure of parallel poly-N-acetylglucosamine chains without intersheet H-bonds. The parallel arrangement of chitosan/1,3-β-d-glucan polymer chains allows for more flexibility than the antiparallel configuration; in consequence, the resultant matrices possess an immense strength [56,57]. Based on spectroscopic data, it can be concluded that strong physical bonds may be created between the following functional groups: COO^−^, NH_2_, NHCOCH_3_ and OH moieties. In samples subjected to 90 °C, hydrophobic interactions probably occurred between the CH_3_ groups of the D-glucosyl residues on 1,3-β-d-glucan units. Therefore, increasing the cross-linking degree of these mixed polysaccharide matrices with increasing reaction temperature was again admitted [12]. Additionally, the spectral data retrieved confirm a higher ratio of intramolecular hydrogen bonds in the 90 °C sample compared to films gelled at lower temperatures. This is confirmed by the results of spectral deconvolution, the calculated coefficients, especially the low I_C–O–C_/I_C–C_ ratio and the high A_amide I_/A_amide II_ ratio, and the percentage of C, O, N atoms on the surface obtained from XPS data.

Within the field of polymer sciences, AFM has been utilized to determine the elasticity of polymer chains, chain conformation, molecular stiffness of hyperbranched macromolecules, and the distribution of single components of polymers on the surface [58]. The disappearance of the fibrous structure of glucan and the grain-like structure of chitosan and the creation of a uniform and homogenous structure of hybrid chitosan/1,3-β-d-glucan matrices testifies to the interconnection of both components and the high strength of mutual interactions. However, randomly distributed protrusions and the presence of a small number of minor grains can provide an excellent base for cell adhesion. It was proven that cells are the most inclined to proliferate on polar, positively charged and slightly rough surfaces [59]. Such conditions are perfectly fulfilled by the hybrid chitosan/1,3-β-d-glucan films.

The results obtained with all used analytical techniques indicate the hybrid nature of the created polymer consisting of chitosan and 1,3-β-d-glucan. It means that the physico-chemical characteristics of the tested material result not only from the sum of the properties of individual components but from the completely new properties that arise [60]. This is due to the strong covalent, ionic and noncovalent bonds formed between the particular constituents.

The cytotoxicity assessment performed against human normal skin fibroblasts (BJ cell line) in direct contact with the chitosan/1,3-β-d-glucan film proved its non-cytotoxicity, indicating its potential in biomedical applications.

The chitosan/1,3-β-d-glucan matrices open a new approach of application in biomedical sciences that may be employed for soft tissue engineering, in particular for skin wound healing and drug delivery systems [61]. These films are capable of forming a complex with oppositely charged ionic polymers, and these interactions have characteristics of poly-cationic/polyanionic complexation. Thanks to the stronger intersheet, hydrogen and ionic bonds are thermally stable [61]. Considering the obtained physico-chemical properties, we can conclude that the gelation temperature of 90 °C seems to be the most favorable from the biomedical engineering point of view. This could be a promising biomaterial used in medicine in the future.

## 4. Materials and Methods

### 4.1. Formation of Hybrid Chitosan/1,3-β-d-Glucan Matrices

The hybrid chitosan/1,3-β-d-glucan film was composed of 2% krill chitosan (1174 kDa and 73% deacetylated, Sea Fisheries Research Institute in Gdynia, Gdynia, Poland) and 8% 1,3-β-d-glucan known as curdlan (Wako Pure Chemicals Industries, Osaka, Japan). To prepare the biomaterial, 4% (*w*/*v*) chitosan was suspended in 1% (*v*/*v*) acetic acid solution, and 16% (*w*/*v*) curdlan was suspended in distilled water. In the next step, both suspensions were thoroughly mixed, keeping the ratio 1:1. They were then spread on the glass coverslip and heated for 20 min in a water bath at 90 °C. After the thermal gelation samples were cooled and neutralized in sodium hydroxide (Avantor Performance Materials, Gliwice, Poland), the samples were finally washed in distilled water and air-dried. The samples of pure chitosan and 1,3-β-d-glucan (as a reference) were produced with the application of an identical procedure. Specimens were stored in the presence of a moisture absorbent (silica gel).

### 4.2. Attenuated Total Reflection Fourier Transform Infrared (ATR FT-IR) Spectroscopy

Infrared spectra were collected with the application of an FT-IR Nicolet 8700 Continuum (Thermo Scientific, Waltham, MA, USA) spectrometer in the attenuated total reflection (ATR) mode utilizing a GladiATR crystal (PIKE Technologies Inc., Madison, WI, USA). Spectra were recorded with the use of Omnic 8 software from Thermo Fisher Scientific (Madison, WI, USA). All ATR FT-IR spectra were measured at room temperature in the 4000–750 cm^−1^ range. Each spectrum represented an average of 5 spectra after baseline correction; every spectrum consisted of a mean of 120 scans. The spectral resolution was 4 cm^−1^. Samples were constantly purged with compressed air during the measurement in order to remove humidity.

### 4.3. Raman Spectroscopy

The Raman spectra were collected applying a DXR confocal Raman Microscope with a three-axis X-Y-Z micropositioner stage connected with the Omnic 8 software from Thermo Fisher Scientific (Madison, WI, USA). A Peltier-cooled CCD detector recording dispersed light was utilized. The excitation laser wavelength was 532 nm. All spectra were measured in the 350–2000 cm^−1^ spectral range with an operating spectral resolution of 4 cm^−1^ of Raman shift, 16 exposures per point in time of 10s and the laser power set to 9 mW. A 50 μm pinhole aperture and 0.5 min photobleaching time were selected. Each spectrum represented an average of 5 spectra after a baseline correction.

### 4.4. Second-Order Derivative Determination, Spectral Deconvolution and Ratios Calculations

To assess the molecular arrangements in the examined samples, second-order derivative spectra were determined after smoothening (Savitzky–Golay algorithm with nine points). Deconvolution of the overlapping bands into individual subbands involved the mixed Lorentzian/Gaussian peak fitting (Voight function) up until the root-mean-square (RMS) value was <0.035 and iterating until the change in weighted sums of squared residuals WSSR was <0.0001 and FWHH <3. This operation was carried out after ATR FT-IR spectra normalization to 1650, 1641, and 1646 cm^−1^ in the 1750–1500 cm^−1^ range, to 1374, 1368, and 1371 cm^−1^ in the 1500–1180 cm^−1^ region, and to 1026, 1029, and 1027 cm^−1^ in the 1180–800 cm^−1^ bandwidth for chitosan, 1,3-β-d-glucan and chitosan/1,3-β-d-glucan, respectively. The Raman spectra were normalized to 1376, 1364, and 1362 cm^−1^ in the 1260–1500 cm^−1^ range, and 1115, 1118, and 1116 cm^−1^ in the 950–1200 cm^−1^ region for chitosan, 1,3-β-d-glucan and chitosan/1,3-β-d-glucan, accordingly. The content of each compound is presented as a percentage by dividing the area of the respective band component by the sum of the entire band area. FT-IR absorbance (A_amide I/Aamide II_, A_CH2_/A_CH3_, A_C=O_/A_C–O_) and Raman intensity ratios (I_CH and/or C–OH_/I_CH3_, I_C–C_/I_C–O–C_) were calculated from 5 single spectra after normalization to 1026, 1029, and 1027 cm^−1^ (FT-IR spectra), and 1099, 1095, and 1092 cm^−1^ (Raman spectra) for chitosan, 1,3-β-d-glucan and chitosan/1,3-β-d-glucan, respectively. The results are shown as mean ± standard deviation. All of the spectral operations were performed in GRAMS/AI software (ThermoGalactic Industries, Keene, NH, USA).

### 4.5. X-ray Photoelectron Spectroscopy (XPS)

X-ray photoelectron spectroscopy (XPS) measurements were carried out with the use of a Microlab 350 (Thermo Electron Corporation, Beverly, MA, USA) spectrometer with non-monochromatic Al Kα radiation (hν = 1486.6 eV) as an X-ray source operating at 2 × 5 mm spot size, 300 W and 15 kV. The high-resolution (HR) XPS spectra were recorded by a hemispherical analyzer at a pass energy of 40 and an energy step size of 0.1 eV. In order to evaluate the XPS data, the Thermo Scientific Avantage software (version 4.88, Waltham, MA, USA) was applied. Deconvolution of HR XPS spectra was performed by applying a smart-type background and a Gaussian peak shape with 35% Lorentzian character. The measured binding energies were corrected relative to the energy of carbon peak (C1s) at 284.6 eV.

### 4.6. Atomic Force Microscopy (AFM)

Atomic force microscopy (Dimension Icon AFM, Bruker, Billerica, MA, USA) in tapping mode using standard AFM probes was applied to visualize the surface topography of the samples at a high resolution. The microscopic measurements were repeated for diverse surface areas of the samples to gather a set of information and perform statistical analysis. The shown images are representative of the studied samples.

### 4.7. Cytotoxicity Evaluation

A cytotoxicity assessment against human normal skin fibroblasts (BJ cell line) was performed in direct contact with the chitosan/1,3-β-d-glucan film. The BJ cell line was obtained from American Type Culture Collection (ATCC-LGC Standards, Teddington, UK). The BJ cells were cultured in Eagle’s minimum essential medium (EMEM, ATCC-LGC Standards, Teddington, UK) containing 10% fetal bovine serum (Pan-Biotech GmbH, Aidenbach, Bavaria, Germany), 100 U/mL penicillin, and 0.1 mg/mL streptomycin (Sigma-Aldrich Chemicals, Warsaw, Poland) and maintained at 37 °C in a humidified atmosphere (95% air, 5% CO_2_). Before cell seeding, the film was cut into small square samples (6 mm × 6 mm), stuck with agarose solution to the bottom of the wells of 48-multiwell plate, and pre-soaked overnight in the complete culture medium. Then, BJ cells at a concentration of 1 × 10^5^ cells were seeded onto the surface of the films and cultured for 48 h. The viability of BJ cells was determined by using the Live/Dead Double Fluorescent Staining Kit (Sigma-Aldrich Chemicals, Warsaw, Poland) and followed by a confocal laser scanning microscope (CLSM, Olympus Fluoview equipped with FV1000, Olympus Corporation, Tokyo, Japan) visualization.

## 5. Conclusions

In this study, we have successfully fabricated and analyzed the physico-chemical properties of two-component hybrid chitosan/1,3-β-d-glucan films gelled at 90 °C. Vibrational spectroscopic (ATR FT-IR and Raman spectroscopy) and XPS spectroscopy studies showed that the surfaces of the hybrid chitosan/1,3-β-d-glucan polymeric matrices cross-linked at 90 °C are wealthy in N, O and especially C atoms, pointing to the involvement of the C-C and C-H bonds and C=O⋯HN moieties in the polymerization process. Additionally, AFM microscopy visualized uniform and homogenous morphology of the sample with only small protrusions. All these results indicate strong chemical interactions between the individual components, i.e., chitosan and 1,3-β-d-glucan in the hybrid matrix.

Based on the spectroscopic and microscopic studies presented, as well as our cytotoxicity evaluation, we expect that the examined biomaterial will induce proper both in vitro and in vivo cellular response due to its satisfactory surface parameters. The results of this research demonstrated the possibility of using polysaccharide-based polymeric scaffolds as an alternative to actual regenerative medicine treatments. It was also proven that spectroscopic combined with microscopic methods may be used as cost-efficient, rapid, and no-preparation tools to determine the molecular structure of biopolymers and provide chemical and morphological analysis of the surface and, in consequence, allow their utility in biomedicine to be assessed.

## Figures and Tables

**Table 1:** The most prominent bands detected in ATR FT-IR spectra of chitosan, 1,3-β-d-glucan, and chitosan/1,3-β-d-glucan films cross-linked at 90 °C.

Wavenumber/cm^−1^			The Type of Vibration and Assignment *
Chitosan	1,3-β-d-Glucan	Chitosan/ 1,3-β-d-Glucan 90 °C	
3353	-	-	ν (OH) of glucopyranose units
3289	3300	3291	ν (OH), water
3085	-	-	ν (N–H)
2914	-	-	ν (CH_2_)
2871	2884	2881	ν (C–H)
1650	1641	1646	80% ν (C=O), 20% ν (C–N) in chit and chit/glu, τ (HOH), amide I, water
1566	-	1584	60% τ (N–H), 30% ν (C–N), 10% ν (C–C), amide II
-	-	1454	δ_s_ (CH_3_), δ_s_ (CH_2_)
1419	1421	1421	δ_s_ (CH_3_), δ_s_ (CH_2_), δ_s_ (CH), ν_s_ (C=O)
1374	1368	1371	δ_s_ (CH_3_)
1313	1312	1312	δ (C–H), δ (CH_2_) δ (OH), N-acetylglucosamine (chitosan), amide III
1259	1252	1259	ν (C–O), ν (N–H) in chit and chit/glu, ν (C–O–C), ν (C–OH)
-	-	1241	ν (C–H) in rings
1198	1203	1202	ν CH_2_OH
1150	1156	1155	τ (C–H), ν_as_ (C–O), ν (C–N) in chit and chit/glu, ν_as_ (C–O–C) in β-glycosidic linked rings
-	1106	1108	ν (C–C), ν (C–O)
1061	1066	1066	ν (C–O)
1026	1029	1027	ν skeletal (C=O)
992	992	992	δ (C=O), τ (C–C)
949	-	-	trans ν (C=C)
894	887	889	δ (C–H), β-glycosidic bonds

(*) Vibration assignment: stretching vibrational mode (ν), deformational (δ); bending (τ), and symmetrical (s) and antisymmetrical (as) modes.

**Table 2:** The most prominent bands detected in the Raman spectra of chitosan, 1,3-β-d-glucan, and chitosan/1,3-β-d-glucan films cross-linked at 90 °C.

Raman Shift/cm^−1^			The Type of Vibration and Assignment *
Chitosan	1,3-β-d-Glucan	Chitosan/ 1,3-β-d-Glucan 90 °C	
289	302	293	δ (C–C–C)
329	319	324	δ (C–C–C)
-	-	337	τ (O–C–O)
376	350	357	δ (C–C–C)
-	399	397	δ C–C(=O)C
423	427	425	τ _out of plane_ (H–C–C=O)
440	-	-	*ν*_s_ (NH_2_) in rings
485	472	467	τ (N–C=O) in chit and chit/glu, τ _in plane_ (C–O–C)
-	-	511	τ _in plane_ (C–O–C)
-	527	527	δ (C–C–N) in chit/glu, δ (C–C–C)
576	570	579	δ (C–C–C)
-	611	608	τ _out of plane_ (C–H)
-	-	562	τ (C–C=O)
-	-	829	δ aromatic (C–N=C)
896	890	892	δ _out of plane_ (C–H), β-glycosidic bond
-	-	919	ν_s_ (C–O–C)
-	-	938	δ _out of plane_ (C–H)
948	-	954	ν (C–H) in rings
-	984	976	ring skeleton stretching vibrations sensitive to anomeric structure of glucose
-	-	997	ν (C–O–C)
1043	1037	1034	δ (C–H), (C–C), (C–OH)
-	-	1063	ν (C–O–C) in rings
1099	1095	1092	ν_s_ (C–O–C) in rings
1115	1118	1116	ν_as_ (C–O–C), ether
1149	1142	1146	ν (C–N) in chit and chit/glu, ν (C–C)
1200	1205	1202	ν (C–CH)
1229	1238	1228	ν (C–H) in rings
1266	1266	1253	δ _in plane_ (C–H), CH_2_OH
-	1319	1315	δ _in plane_ (C–H)
1346	-	-	τ (C–H)
1376	1364	1362	ν (C–N) in chit and chit/glu, ν (C–H), ν (C–OH)
1413	1412	1410	δ_as_ (CH_3_)
-	-	1422	ν (C–C)
1460	1459	1461	δ_as_ (C–H), τ _in plane_ (CH_2_)
1661	-	-	ν (C=O), amide I
1856	-	-	ν (C–C), τ (C=O)

(*) Vibration assignment: stretching vibrational mode (ν), deformational (δ); bending (τ), and symmetrical (s) and antisymmetrical (as) modes.

**Table 3:** Average calculated values of the ATR FT-IR absorbance and Raman intensity ratios for particular chemical groups detected in studied films. The results are presented as mean ± standard deviation.

The FT-IR Absorbance Ratio	Sample		
	Chitosan *	1,3-β-d-Glucan **	Chitosan/ 1,3-β-d-Glucan 90 °C ***
A_amide I/_A_amide II_	1.200 ± 0.068	–	1.490 ± 0.048
A_CH__2__/_A_CH__3_	0.838 ± 0.015	0.737 ± 0.035	0.834 ± 0.025
A_C=O/_A_C_–_O_	0.997 ± 0.026	1.228 ± 0.036	1.131 ± 0.016
**The Raman intensity ratio**			
I_CH and/or C_–_OH/_I_CH__3_	1.819 ± 0.156	2.008 ± 0.039	1.844 ± 0.067
I_C_–_O_–_C/_I_C_–_C_	1.944 ± 0.129	1.844 ± 0.067	1.451 ± 0.058

* FT-IR spectroscopy: amide I 1650 cm^−1^, amide II 1556 cm^−1^, CH_2_ 1313 cm^−1^, CH_3_ 1374 cm^−1^, C=O 992 cm^−1^, C–O 1061 cm^−1^; Raman spectroscopy: CH and/or C–OH 1376 cm^−1^, CH_3_ 1413 cm^−1^, C–O–C 1115 cm^−1^, C–C 1149 cm^−1^; ** FT-IR spectroscopy: amide I 1641 cm^−1^, amide II –, CH_2_ 1312 cm^−1^, CH_3_ 1368 cm^−1^, C=O 992 cm^−1^, C–O 1066 cm^−1^; Raman spectroscopy: CH and/or C–OH 1364 cm^−1^, CH_3_ 1412 cm^−1^, C–O–C 1118 cm^−1^, C–C 1142 cm^−1^; *** FT-IR spectroscopy: amide I 1646 cm^−1^, amide II 1584 cm^−1^, CH_2_ 1312 cm^−1^, CH_3_ 1371 cm^−1^, C=O 992 cm^−1^, C–O 1066 cm^−1^; Raman spectroscopy: CH and/or C–OH 1362 cm^−1^, CH_3_ 1410 cm^−1^, C–O–C 1116 cm^−1^, C–C 1146 cm^−1^.

**Table 4:** C1s_,_ O1s and N1s binding energies (BE, eV) evaluated from a deconvolution procedure of corrected XPS spectra for glucan, chitosan and chitosan/1,3-β-d-glucan samples.

	Binding Energy/eV High Resolution Spectra					Chemical Composition
Glucan	C1s	O1s	N1s	C:O At. % ratio (C:N)	species	
	284.6			2.15	C-C/C-H	C—64.3
	286.3	532.8	399.7	−13.39	C-OH, C-N	O—29.9
	287.6	531.3			O-C-O, C=O	N—4.8
	282.4				Si-C-O	Na—0.0
		535.8			H_2_O	Si—0.3
			401.6		C-NH_x_	Ca—0.0
						S—0.8
						P—0.0
						Pb—0.0
						Cl—0.0
Chitosan	C1s	O1s	N1s	C:O At. % ratio (C:N)	species	
	284.6			2.58	C-C/C-H	C—67.2
	286.1	532.7	399.9	−12.24	C-OH, C-N	O—26.0
	287.9	531.1			O-C-O, C=O	N—4.9
	282.3				Si-C-O	Na—0.8
		535.5			H_2_O	Si—0.0
			401.9		C-NH_x_	Ca—0.0
						S—0.4
						P—0.0
						Pb—0.0
						Cl—0.4
Chitosan/β-1,3-glucan	C1s	O1s	N1s	C:O At. % ratio (C:N)	species	
	284.6			2.32	C-C/C-H	C—62.9
	286.1	532.7	399.9	−13.81	C-OH, C-N	O—26.1
	287.9	531.1			O-C-O, C=O	N—4.5
	282.3				Si-C-O	Na—3.5
		535.5			H_2_O	Si—0.8
			401.9		C-NH_x_	Ca—0.7
						S—0.8
						P—0.4
						Pb—0.0
						Cl—0.2

## Data Availability

The raw/processed data required to reproduce these findings can be obtained from the corresponding authors upon reasonable request.

## Supplementary Materials

The following supporting information can be downloaded at: https://www.mdpi.com/article/10.3390/ijms23115953/s1.
