# Supplementary material for: Surface Chemical and Morphological Analysis of Chitosan/1,3-β-d-Glucan Polysaccharide Films Cross-Linked at 90 °C
Source: Int J Mol Sci. 2022 May 25;23(11):5953. doi: 10.3390/ijms23115953 (PMC9180171; doi:10.3390/ijms23115953)
Supplement: Supplementary file 1 [file ijms-23-05953-s001.zip › ijms-1696275-supplementary.pdf]

Table S1. The most prominent bands detected in ATR FT-IR spectra of chitosan, 1,3-β-D-glucan, and chitosan/1,3-β-D-glucan films cross-linked at 90°C and at intermediate temperatures.

Raman shift / cm^{-1}			Wavenumber / cm^{-1}		
**chitosan/ 1,3-β-D-glucan 90°C**	**chitosan/ 1,3-β-D-glucan 70-80°C**	**The type of vibrations and assignment***	**chitosan/ 1,3-β-D-glucan 90°C**	**chitosan/ 1,3-β-D-glucan 70-80°C**	**The type of vibrations and assignment***
293	293	δ(C–C–C)	889	889	δ(C–H), β-glycosidic bonds
324	324	δ(C–C–C)	992	992	δ(C=O), τ(C–C)
337	-	τ(O–C–O)	1027	1027	ν skeletal (C=O)
357	357	δ(C–C–C)	1066	1066	ν(C–O)
397	397	δ(C–C(=O)C	1108	-	ν(C–C), ν(C–O)
425	425	$\tau_{out\ of\ plane}$ (H–C–C=O)	1155	1155	τ(C–H), ν_{as}(C–O), ν(C–N), ν_{as}(C–O–C) in β-glycosidic linked rings
467	-	τ(N–C=O), $\tau_{in\ plane}$ (C–O–C)	1202	1202	νCH_2OH
511	511	$\tau_{in\ plane}$ (C–O–C)	1241	-	ν(C–H) in rings
527	527	δ(C–C–N), δ(C–C–C)	1259	1259	ν(C–O), ν(N–H), ν(C–O–C), ν(C–OH)
562	-	τ(C–C=O)	1312	1312	δ(C–H), δ(CH_2) δ(OH), N-acetylglucosamine (chitosan), amide III
579	579	δ(C–C–C)	1371	1371	$\delta_s(CH_3)$
608	608	$\tau_{out\ of\ plane}$ (C–H)	1421	1421	$\delta_s(CH_3)$, $\delta_s(CH_2)$, δ_s(CH), ν_s(C=O)
829	829	δ aromatic (C–N=C)	1454	1454	δ_s (CH_3), $\delta_s(CH_2)$
892	892	$\delta_{out\ of\ plane}$ (C–H), β-glycosidic bond	1584	1566	60% τ(N–H), 30% ν(C–N), 10% ν (C–C), amide II
919	-	ν_s(C–O–C)	1646	1646	80% ν(C=O), 20% ν(C–N), τ(HOH), amide I, water
938	938	$\delta_{out\ of\ plane}$ (C–H)	2881	2881	ν(C–H)
954	954	ν(C–H) in rings	-	2924	ν(CH_2)
976	-	ring skeleton stretching vibrations sensitive to anomeric structure of glucose	3291	3291	ν(OH), water
997	997	ν(C–O–C)			
1034	1032	δ(C–H), (C–C), (C–OH)			
1063	1061	ν(C–O–C) in rings			
1092	1092	ν_s(C–O–C) in rings			
1116	1116	ν_{as}(C–O–C), ether			
1146	1146	ν(C–N), ν(C–C)			
1202	1202	ν(C–CH)			
1228	1228	ν(C–H) in rings			

1253	1253	$\delta_{in\ plane}$ (C–H), CH_2OH
1315	1315	$\delta_{in\ plane}$ (C–H)
1362	1369	ν(C–N), ν(C–H), ν(C–OH)
1410	-	$\delta_{as}(CH_3)$
1422	-	ν(C–C)
1461	1461	δ_{as}(C–H), $\tau_{in\ plane}$ (CH_2)
-	1502	ν(C–C)
-	1620	ν(C=O), amide I

*) Vibrations assignment: stretching vibrational mode (ν), deformational (δ); bending (τ), and symmetrical (s) and antisymmetrical (as) modes.
